# Supplementary material for: Phylotypic Diversity of Bacteria Associated with Speleothems of a Silicate Cave in a Guiana Shield Tepui
Source: Microorganisms. 2022 Jul 11;10(7):1395. doi: 10.3390/microorganisms10071395 (PMC9316562; doi:10.3390/microorganisms10071395)
Supplement: Supplementary file 1 [file microorganisms-10-01395-s001.zip › microorganisms-1720595-supplementary.pdf]

Supplementary materials (Figures S1 to S13 and Table S1) of

"Phylotypic diversity of bacteria associated with speleothems of a silicate cave in a Guiana Shield tepui, Venezuela"

by Qi Liu*, Zichen He*, Takeshi Naganuma, Ryosuke Nakai, Luz María Rodríguez, Rafael Carreño and Franco Urbani

* Co-first authors

Corresponding author: takn@hiroshima-u.ac.jp

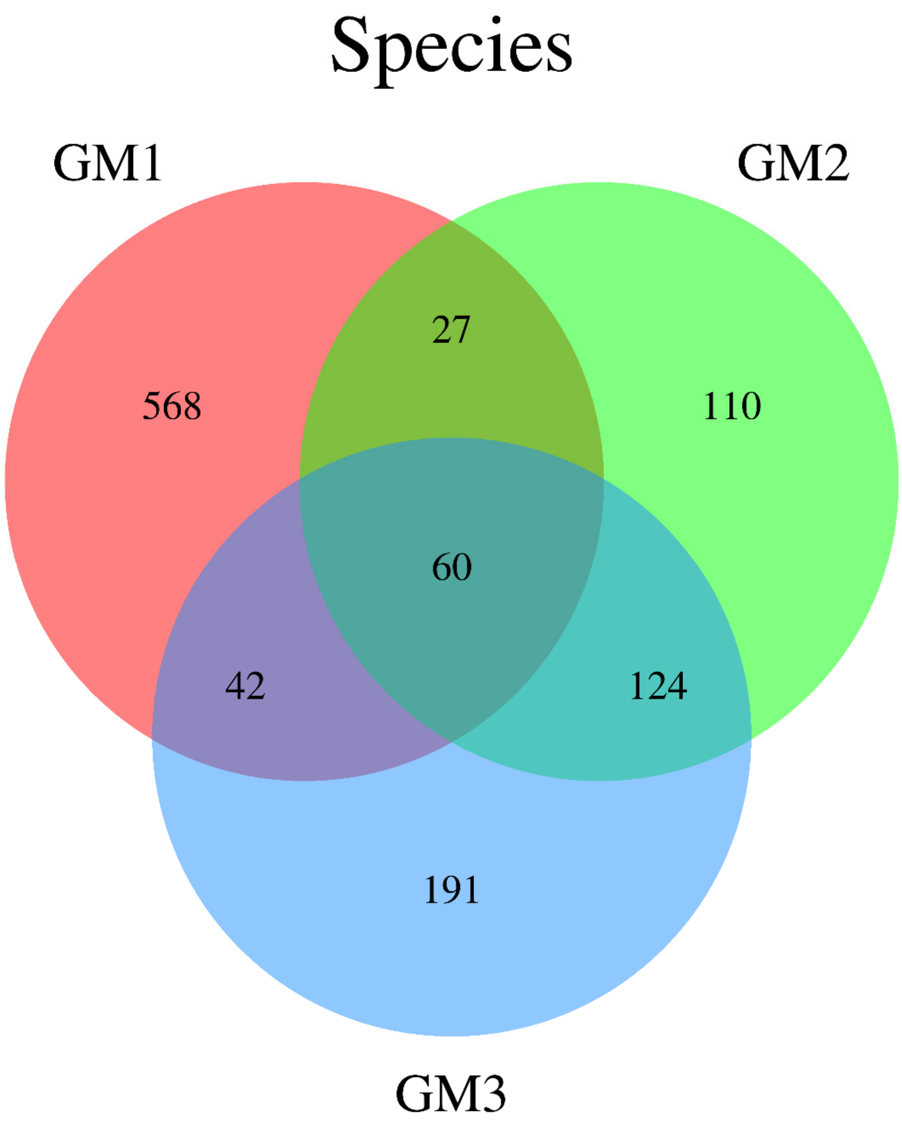

Figure S1. Venn diagram showing the distribution of OTU-affiliated species in GM1, GM2 and GM3 speleothems and their intersections.

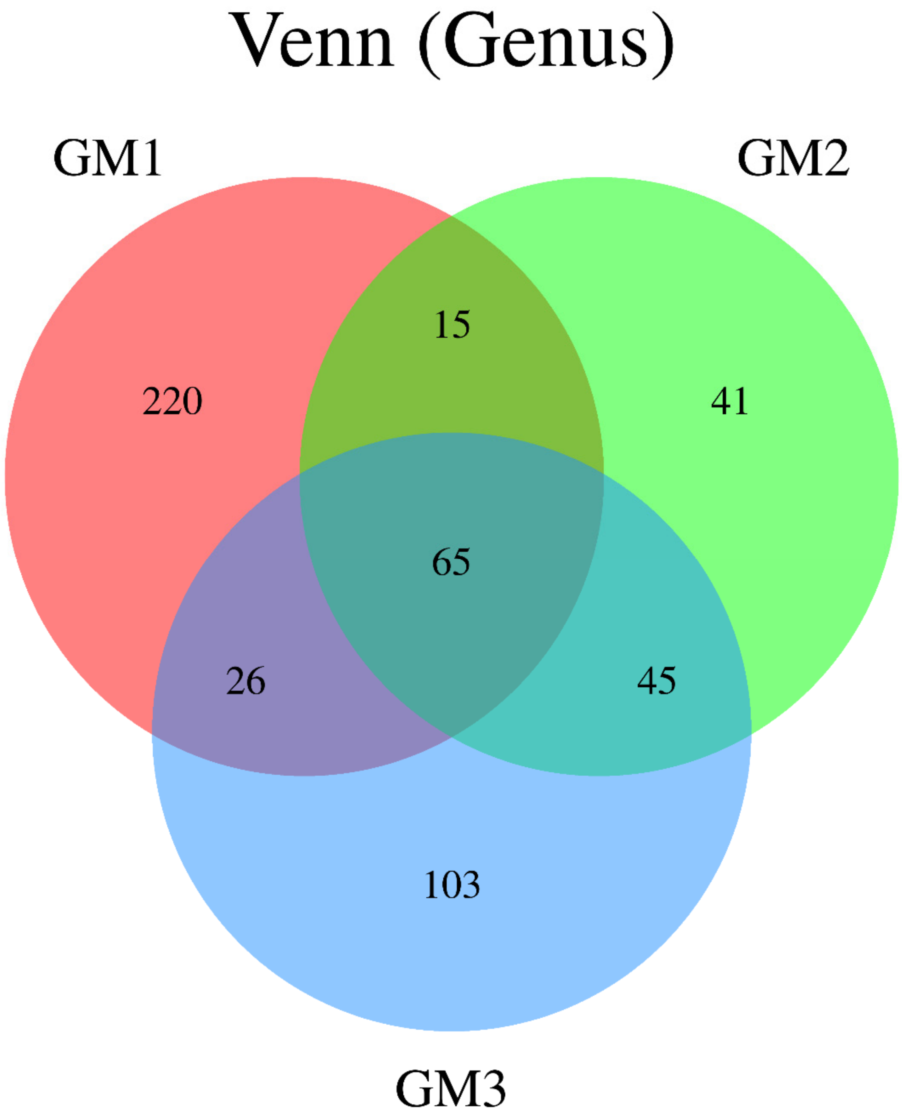

Figure S2. Venn diagram showing the distribution of OTU-affiliated genera in GM1, GM2 and GM3 speleothems and their intersections.

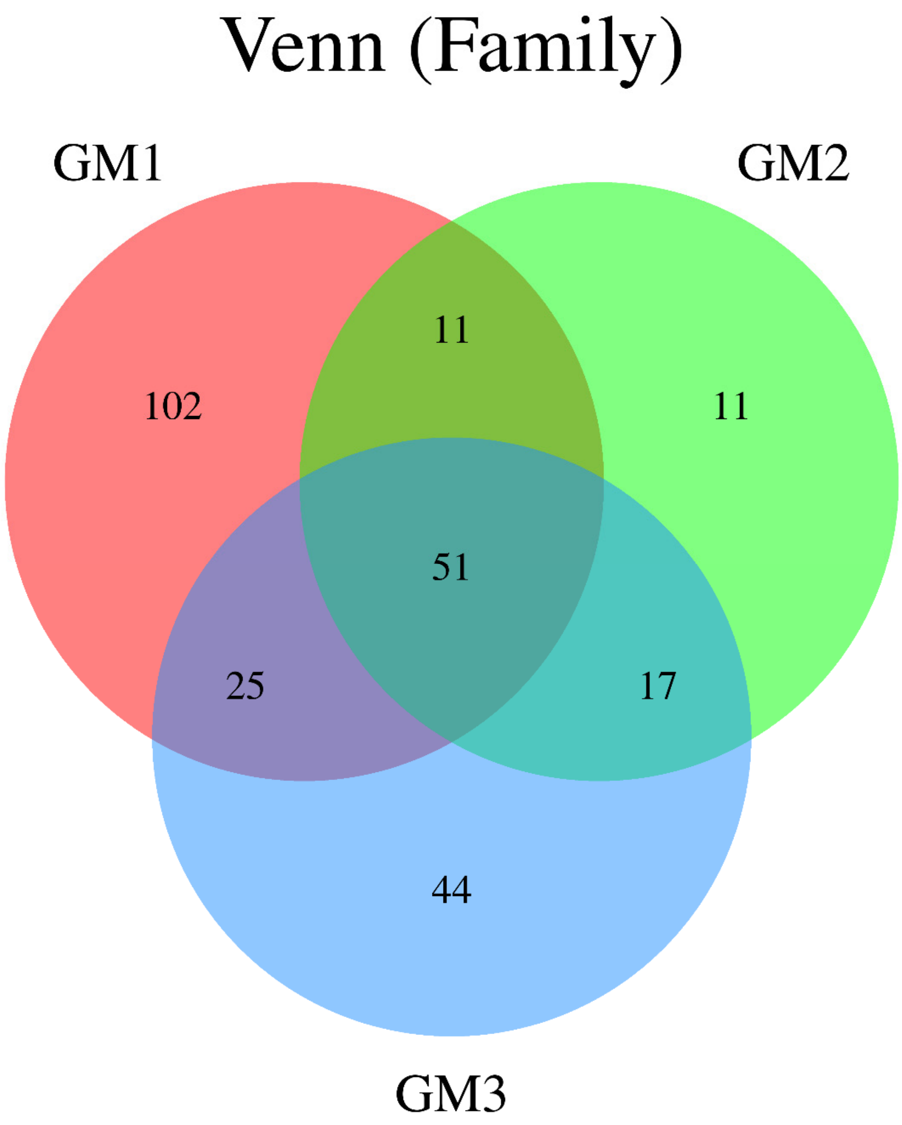

Figure S3. Venn diagram showing the distribution of OTU-affiliated families in GM1, GM2 and GM3 speleothems and their intersections.

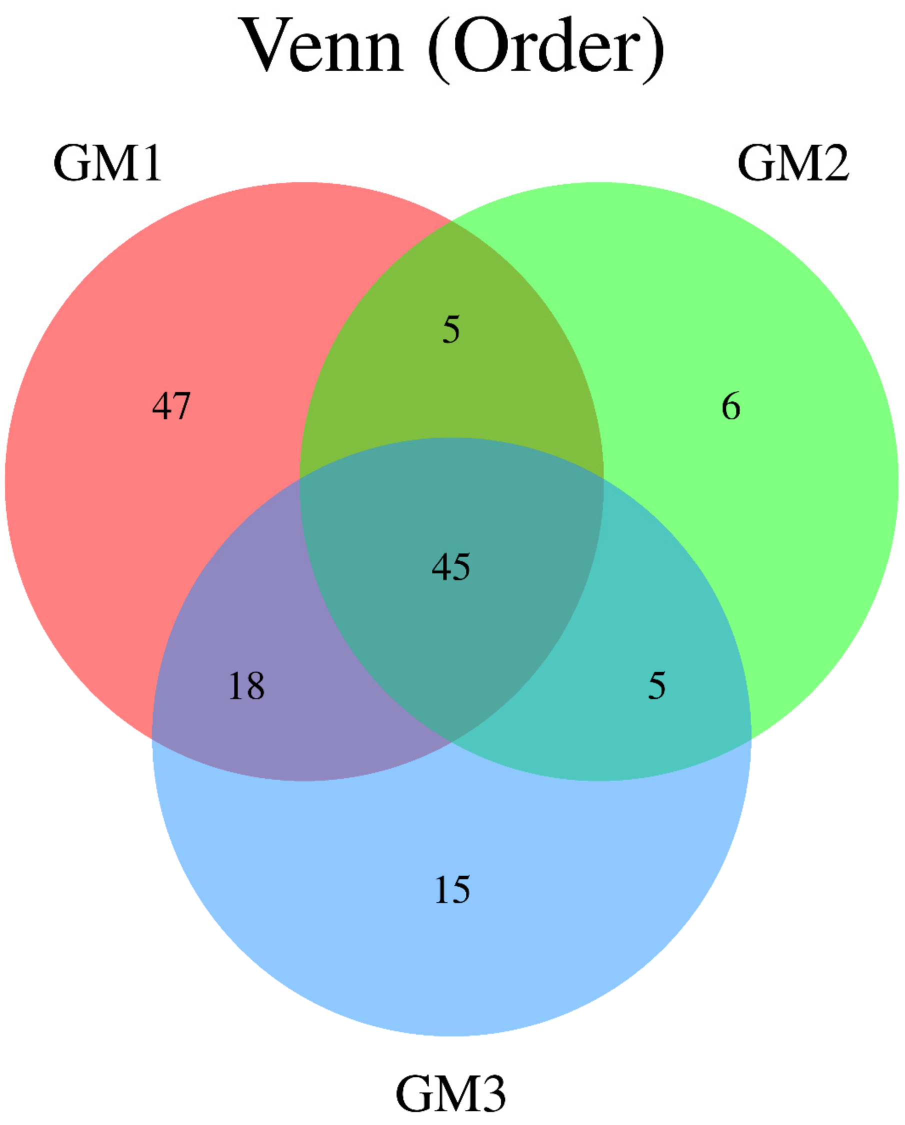

Figure S4. Venn diagram showing the distribution of OTU-affiliated orders in GM1, GM2 and GM3 speleothems and their intersections.

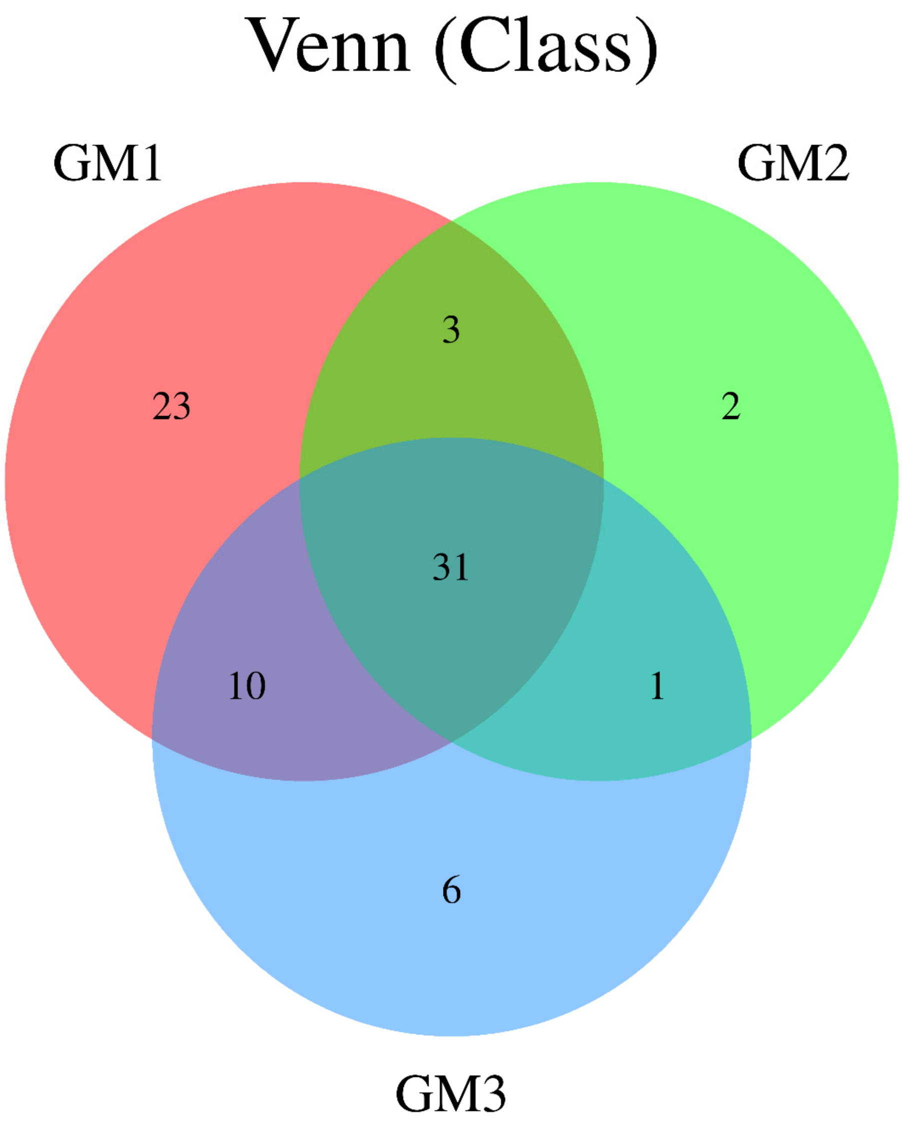

Figure S5. Venn diagram showing the distribution of OTU-affiliated classes in GM1, GM2 and GM3 speleothems and their intersections.

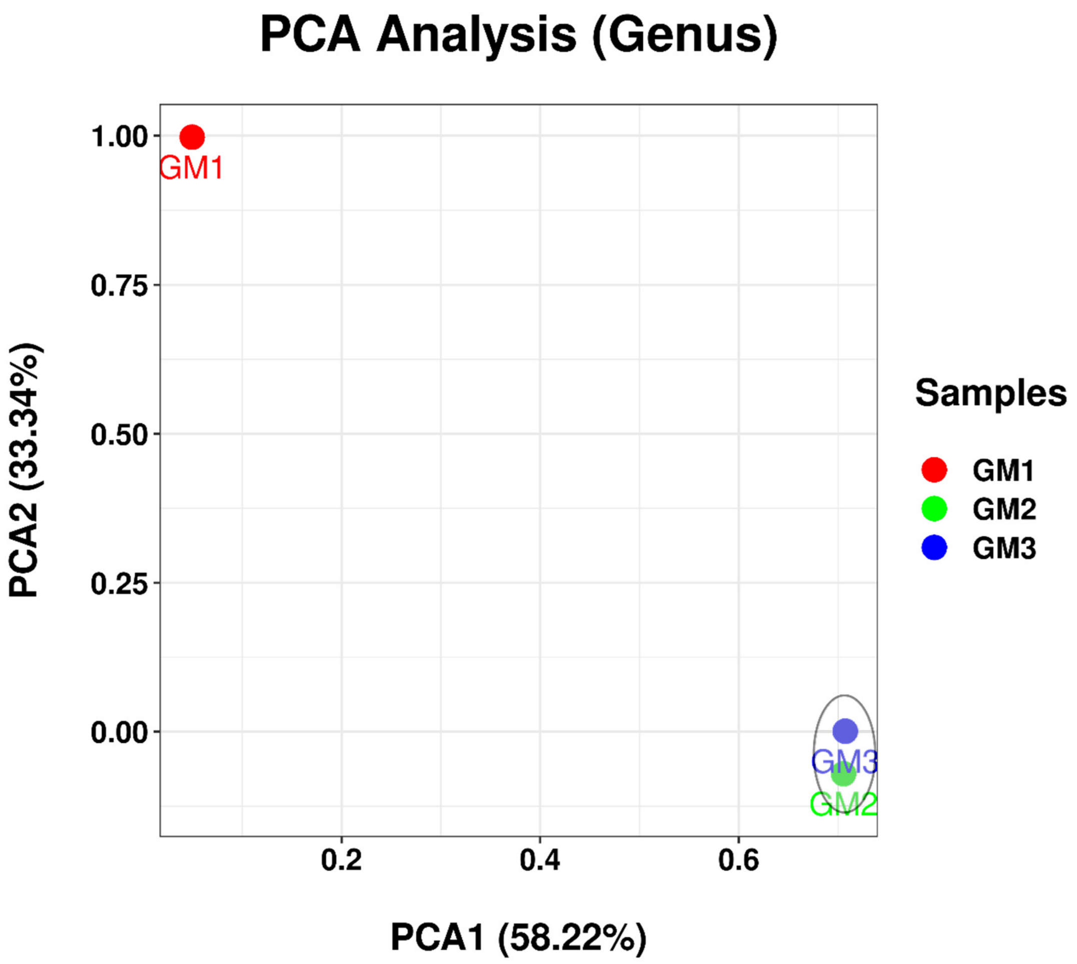

Figure S6. PCA plots of OTU-affiliated genera in the GM1, GM2 and GM3 speleothems.

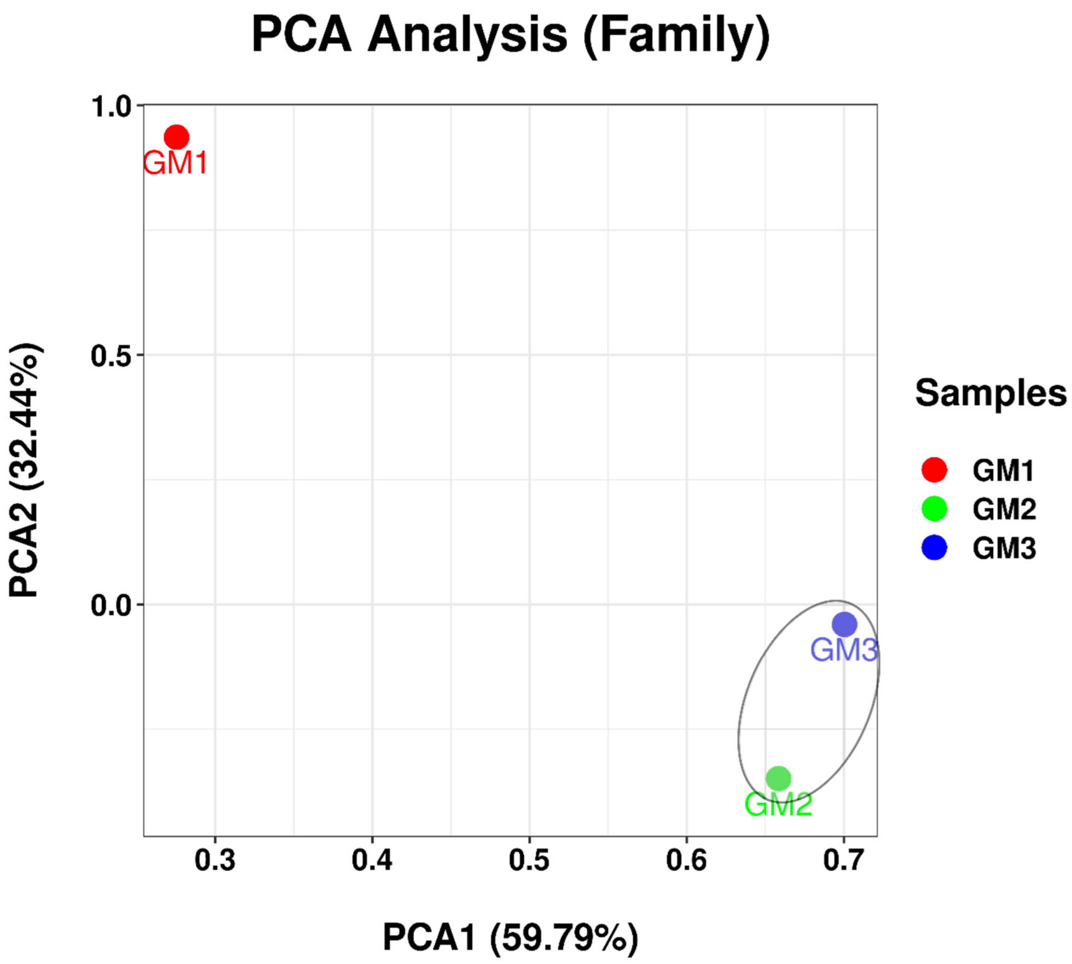

Figure S7. PCA plots of OTU-affiliated families in the GM1, GM2 and GM3 speleothems.

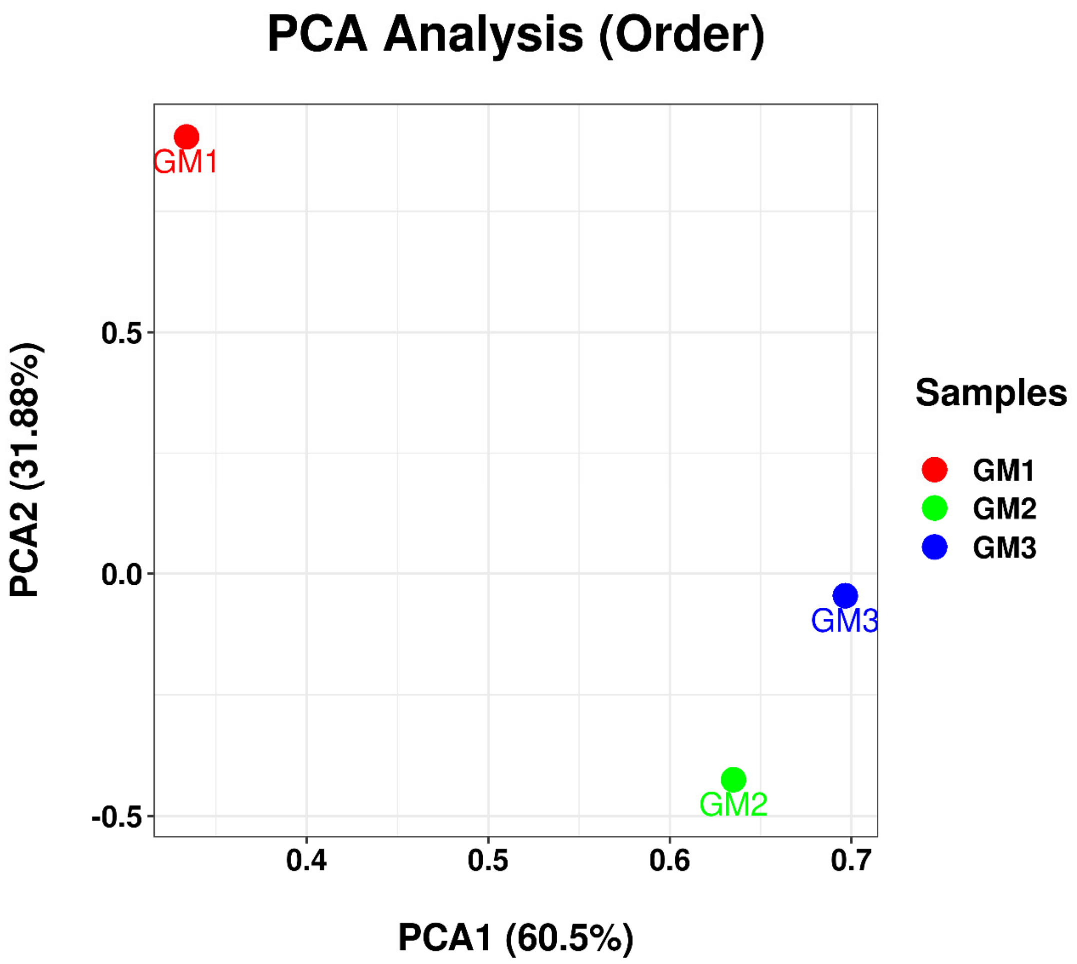

Figure S8. PCA plots of OTU-affiliated orders in the GM1, GM2 and GM3 speleothems.

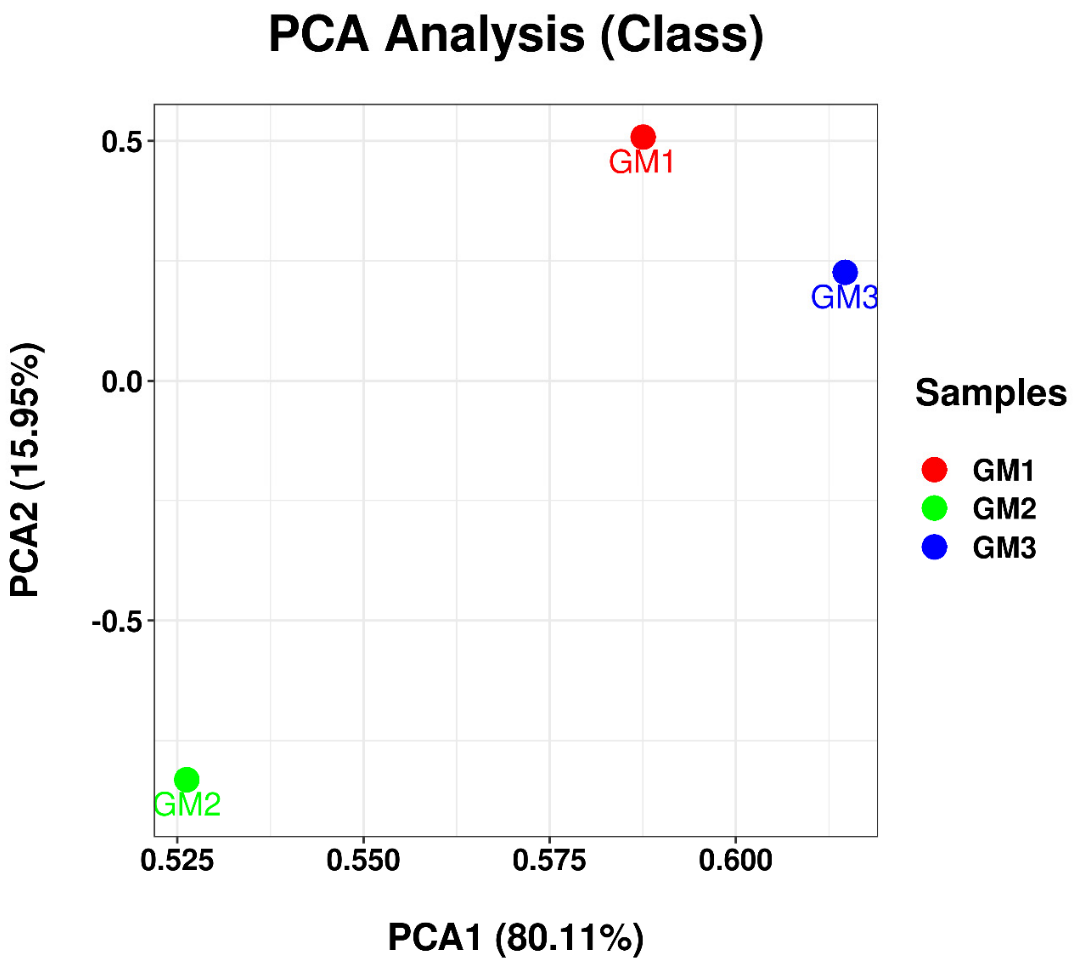

Figure S9. PCA plots of OTU-affiliated classes in the GM1, GM2 and GM3 speleothems.

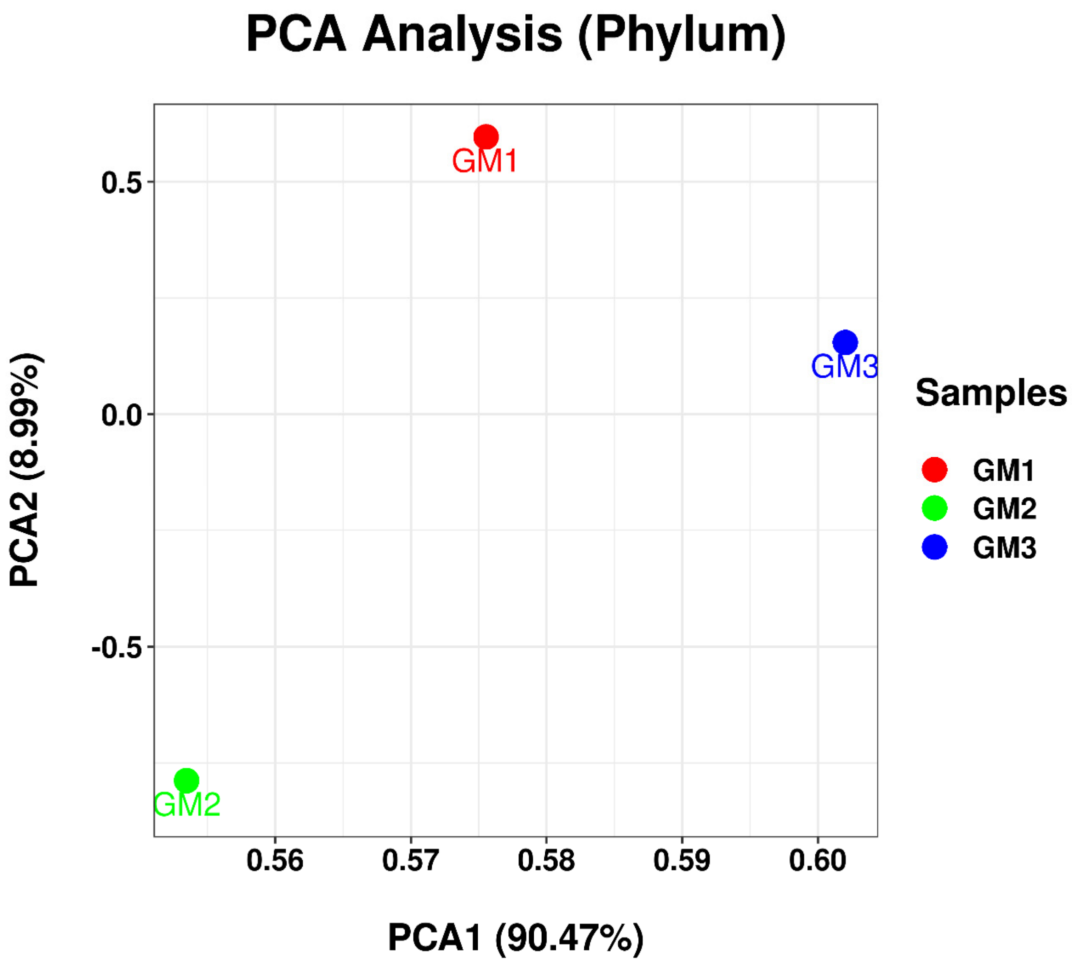

Figure S10. PCA plots of OTU-affiliated phyla in the GM1, GM2 and GM3 speleothems.

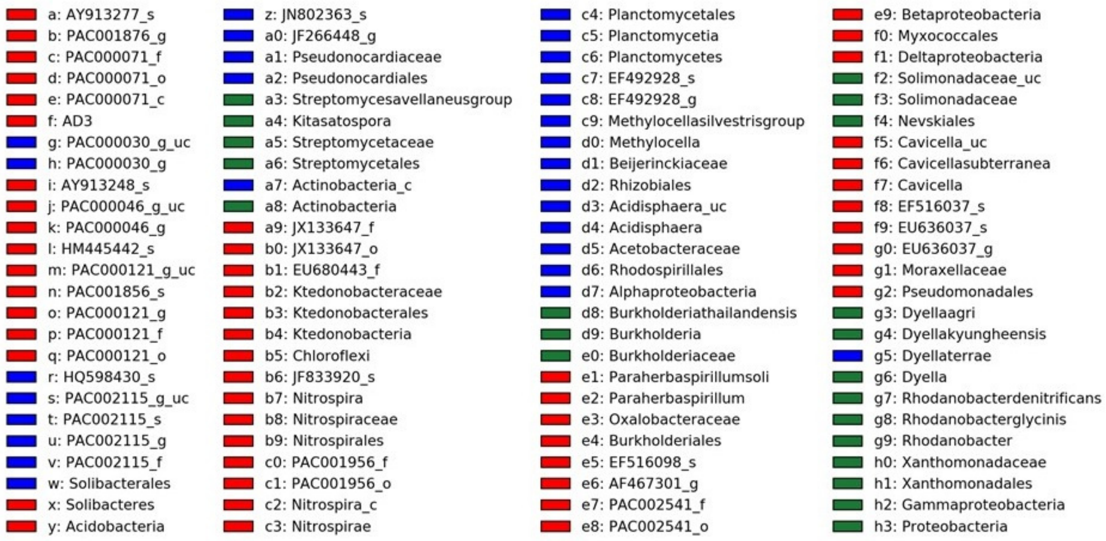

Figure S11. Expedient taxonomic names corresponding to the codes in the LEfSe cladogram (Figure 9 in the main text). More correct taxonomic names/ranks shown in the supplementary Table S1 as well as Table 3 in the main text.

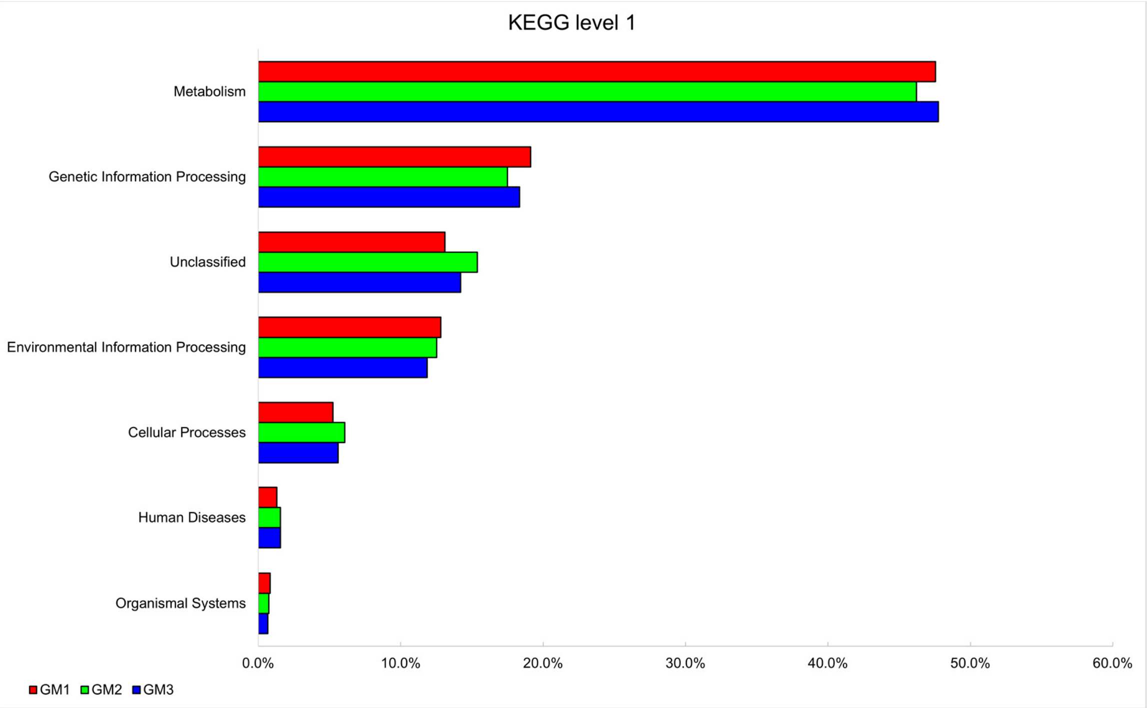

Figure S12. KEGG Level 1 metabolic pathways of GM1 (red), GM2 (green) and GM3 (blue) speleothem microbiomes. Pathways are shown in the order of relative abundances in GM1.

Higher resolution is provided by a separate PNG image
“Churi_FigS13_KEGG_Level-3_all”.

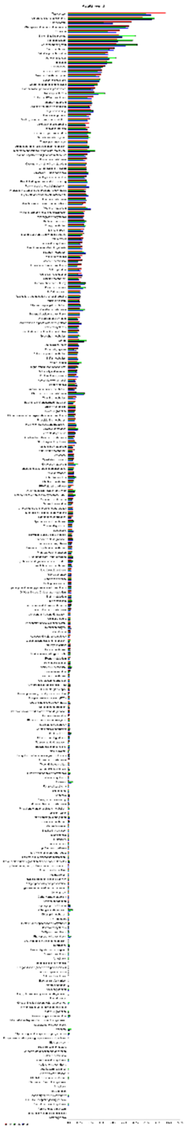

Figure S13. KEGG Level 3 pathways of GM1 (red), GM2 (green) and GM3 (blue) speleothem microbiomes. Pathways are shown in the order of relative abundances in GM1.

Table S1. Taxonomic biomarkers having LDA scores >4 and their corresponding codes in Figures 9 and S11.

Group	Code in Figs. 9 and S11	Biomarker name	LDA value
GM2	h0	Bacteria.Proteobacteria.Gammaproteobacteria.Xanthomonadales.Xanthomonadaceae	5.5023461
GM2	h1	Bacteria.Proteobacteria.Gammaproteobacteria.Xanthomonadales	5.4993716
GM2	g6	Bacteria.Proteobacteria.Gammaproteobacteria.Xanthomonadales.Xanthomonadaceae.Dyella	5.4508021
GM2	h2	Bacteria.Proteobacteria.Gammaproteobacteria	5.3604338
GM2	g4	Bacteria.Proteobacteria.Gammaproteobacteria.Xanthomonadales.Xanthomonadaceae.Dyella.Dyella kyungheensis	5.2825178
GM2	h3	Bacteria.Proteobacteria	5.2760763
GM1	p	Bacteria.Acidobacteria.Solibacteres.PAC000121_o.PAC000121_f	5.2235904
GM1	q	Bacteria.Acidobacteria.Solibacteres.PAC000121_o	5.2217286
GM1	y	Bacteria.Acidobacteria	5.1609085
GM1	x	Bacteria.Acidobacteria.Solibacteres	5.1535597
GM3	g5	Bacteria.Proteobacteria.Gammaproteobacteria.Xanthomonadales.Xanthomonadaceae.Dyella.Dyella terrae	5.0766446
GM3	v	Bacteria.Acidobacteria.Solibacteres.Solibacterales.PAC002115_f	5.0363154
GM3	u	Bacteria.Acidobacteria.Solibacteres.Solibacterales.PAC002115_f.PAC002115_g	5.0205626
GM3	w	Bacteria.Acidobacteria.Solibacteres.Solibacterales	5.0108374
GM1	g1	Bacteria.Proteobacteria.Gammaproteobacteria.Pseudomonadales.Moraxellaceae	4.9736407
GM1	a2	Bacteria.Proteobacteria.Gammaproteobacteria.Pseudomonadales	4.9679824
GM3	d7	Bacteria.Proteobacteria.Alphaproteobacteria	4.9443965
GM1	k	Bacteria.Acidobacteria.Solibacteres.PAC000121_o.PAC000121_f.PAC000046_g	4.9222454
GM1	o	Bacteria.Acidobacteria.Solibacteres.PAC000121_o.PAC000121_f.PAC000121_g	4.9214991
GM2	g3	Bacteria.Proteobacteria.Gammaproteobacteria.Xanthomonadales.Xanthomonadaceae.Dyella.Dyella agri	4.8336432
GM3	d1	Bacteria.Proteobacteria.Alphaproteobacteria.Rhizobiales.Beijerinckiaceae	4.8074057
GM3	d2	Bacteria.Proteobacteria.Alphaproteobacteria.Rhizobiales	4.7880641
GM1	g0	Bacteria.Proteobacteria.Gammaproteobacteria.Pseudomonadales.Moraxellaceae.EU636037_g	4.7445247
GM1	b4	Bacteria.Chloroflexi.Ktedonobacteria	4.7172949
GM1	b5	Bacteria.Chloroflexi	4.7117704

GM3	t	Bacteria.Acidobacteria.Solibacteres.Solibacterales.PAC002115_f.PAC002115_g.PAC002115_s	4.6739806
GM2	g9	Bacteria.Proteobacteria.Gammaproteobacteria.Xanthomonadales.Xanthomonadaceae.Rhodanobacter	4.6703148
GM3	d0	Bacteria.Proteobacteria.Alphaproteobacteria.Rhizobiales.Beijerinckiaceae.Methylocella	4.6700005
GM3	c9	Bacteria.Proteobacteria.Alphaproteobacteria.Rhizobiales.Beijerinckiaceae.Methylocella.Methylocella silvestris group	4.6621020
GM3	g	Bacteria.Acidobacteria.Solibacteres.PAC000121_o.PAC000121_f.PAC000030_g.PAC000030_g_uc	4.6519412
GM3	h	Bacteria.Acidobacteria.Solibacteres.PAC000121_o.PAC000121_f.PAC000030_g	4.6498736
GM2	a8	Bacteria.Actinobacteria	4.6455902
GM1	m	Bacteria.Acidobacteria.Solibacteres.PAC000121_o.PAC000121_f.PAC000121_g.PAC000121_g_uc	4.6383252
GM1	e9	Bacteria.Proteobacteria.Betaproteobacteria	4.6331960
GM3	a7	Bacteria.Actinobacteria.Actinobacteria_c	4.6165807
GM1	b3	Bacteria.Chloroflexi.Ktedonobacteria.Ktedonobacterales	4.5482081
GM1	f7	Bacteria.Proteobacteria.Gammaproteobacteria.Pseudomonadales.Moraxellaceae.Cavicella	4.5375184
GM2	a5	Bacteria.Actinobacteria.Actinobacteria_c.Streptomycetales.Streptomycetaceae	4.5124585
GM3	s	Bacteria.Acidobacteria.Solibacteres.Solibacterales.PAC002115_f.PAC002115_g.PAC002115_g_uc	4.5105809
GM2	a6	Bacteria.Actinobacteria.Actinobacteria_c.Streptomycetales	4.5082925
GM2	a4	Bacteria.Actinobacteria.Actinobacteria_c.Streptomycetales.Streptomycetaceae.Kitasatospora	4.5077466
GM2	a3	Bacteria.Actinobacteria.Actinobacteria_c.Streptomycetales.Streptomycetaceae.Kitasatospora.Streptomyces avellaneus group	4.5020525
GM2	g7	Bacteria.Proteobacteria.Gammaproteobacteria.Xanthomonadales.Xanthomonadaceae.Rhodanobacter.Rhodanobacter denitrificans	4.4695201
GM1	f9	Bacteria.Proteobacteria.Gammaproteobacteria.Pseudomonadales.Moraxellaceae.EU636037_g.EU636037_s	4.4561180
GM1	f8	Bacteria.Proteobacteria.Gammaproteobacteria.Pseudomonadales.Moraxellaceae.EU636037_g.EF516037_s	4.4312256
GM1	e4	Bacteria.Proteobacteria.Betaproteobacteria.Burkholderiales	4.4205926
GM1	c3	Bacteria.Nitrospirae	4.4151632
GM1	c2	Bacteria.Nitrospirae.Nitrospira_c	4.4145500
GM3	d6	Bacteria.Proteobacteria.Alphaproteobacteria.Rhodospirillales	4.4018457
GM1	j	Bacteria.Acidobacteria.Solibacteres.PAC000121_o.PAC000121_f.PAC000046_g.PAC000046_g_uc	4.3575440
GM3	a1	Bacteria.Actinobacteria.Actinobacteria_c.Pseudonocardiales.Pseudonocardiaceae	4.3414036
GM3	d5	Bacteria.Proteobacteria.Alphaproteobacteria.Rhodospirillales.Acetobacteraceae	4.3308215
GM3	a2	Bacteria.Actinobacteria.Actinobacteria_c.Pseudonocardiales	4.3243960
GM1	i	Bacteria.Acidobacteria.Solibacteres.PAC000121_o.PAC000121_f.PAC000046_g.AY913248_s	4.3218226
GM3	d4	Bacteria.Proteobacteria.Alphaproteobacteria.Rhodospirillales.Acetobacteraceae.Acidisphaera	4.3082643

GM1	f6	Bacteria.Proteobacteria.Gammaproteobacteria.Pseudomonadales.Moraxellaceae.Cavicella.Cavicella subterranea	4.3062015
GM3	a0	Bacteria.Actinobacteria.Actinobacteria_c.Pseudonocardiales.Pseudonocardiaceae.JF266448_g	4.2918022
GM1	b1	Bacteria.Chloroflexi.Ktedonobacteria.Ktedonobacterales.EU680443_f	4.2865398
GM1	e3	Bacteria.Proteobacteria.Betaproteobacteria.Burkholderiales.Oxalobacteraceae	4.2430713
GM1	e6	Bacteria.Proteobacteria.Betaproteobacteria.PAC002541_o.PAC002541_f.AF467301_g	4.2280026
GM1	e5	Bacteria.Proteobacteria.Betaproteobacteria.PAC002541_o.PAC002541_f.AF467301_g.EF516098_s	4.2236958
GM2	f2	Bacteria.Proteobacteria.Gammaproteobacteria.Nevskiales.Solimonadaceae.Solimonadaceae_uc	4.2130134
GM2	f4	Bacteria.Proteobacteria.Gammaproteobacteria.Nevskiales	4.2097695
GM1	e7	Bacteria.Proteobacteria.Betaproteobacteria.PAC002541_o.PAC002541_f	4.1961804
GM1	e8	Bacteria.Proteobacteria.Betaproteobacteria.PAC002541_o	4.1942640
GM1	b9	Bacteria.Nitrospirae.Nitrospira_c.Nitrospirales	4.1910190
GM1	f5	Bacteria.Proteobacteria.Gammaproteobacteria.Pseudomonadales.Moraxellaceae.Cavicella.Cavicella_uc	4.1905865
GM2	f3	Bacteria.Proteobacteria.Gammaproteobacteria.Nevskiales.Solimonadaceae	4.1861820
GM1	e	Bacteria.AD3.PAC000071_c	4.1803846
GM1	f	Bacteria.AD3	4.1709352
GM2	g8	Bacteria.Proteobacteria.Gammaproteobacteria.Xanthomonadales.Xanthomonadaceae.Rhodanobacter.Rhodanobacter glycinis	4.1641379
GM1	e2	Bacteria.Proteobacteria.Betaproteobacteria.Burkholderiales.Oxalobacteraceae.Paraherbaspirillum	4.1484142
GM3	c7	Bacteria.Proteobacteria.Alphaproteobacteria.Rhizobiales.Beijerinckiaceae.EF492928_g.EF492928_s	4.1358511
GM1	n	Bacteria.Acidobacteria.Solibacteres.PAC000121_o.PAC000121_f.PAC000121_g.PAC001856_s	4.1347981
GM1	e1	Bacteria.Proteobacteria.Betaproteobacteria.Burkholderiales.Oxalobacteraceae.Paraherbaspirillum.Paraherbaspirillum soli	4.1264952
GM1	b7	Bacteria.Nitrospirae.Nitrospira_c.Nitrospirales.Nitrospiraceae.Nitrospira	4.1249626
GM3	c8	Bacteria.Proteobacteria.Alphaproteobacteria.Rhizobiales.Beijerinckiaceae.EF492928_g	4.1209020
GM1	d	Bacteria.AD3.PAC000071_c.PAC000071_o	4.1178297
GM1	b8	Bacteria.Nitrospirae.Nitrospira_c.Nitrospirales.Nitrospiraceae	4.1169642
GM1	b2	Bacteria.Chloroflexi.Ktedonobacteria.Ktedonobacterales.Ktedonobacteraceae	4.1164177
GM1	c	Bacteria.AD3.PAC000071_c.PAC000071_o.PAC000071_f	4.1144296
GM1	b0	Bacteria.Chloroflexi.Ktedonobacteria.JX133647_o	4.1091602
GM1	f1	Bacteria.Proteobacteria.Deltaproteobacteria	4.1069732
GM1	a9	Bacteria.Chloroflexi.Ktedonobacteria.JX133647_o.JX133647_f	4.0944512
GM1	b6	Bacteria.Nitrospirae.Nitrospira_c.Nitrospirales.Nitrospiraceae.Nitrospira.JF833920_s	4.0731428

GM3	r	Bacteria.Acidobacteria.Solibacteres.Solibacterales.PAC002115_f.PAC002115_g.HQ598430_s	4.0726757
GM3	d3	Bacteria.Proteobacteria.Alphaproteobacteria.Rhodospirillales.Acetobacteraceae.Acidisphaera.Acidisphaera_uc	4.0663275
GM1	f0	Bacteria.Proteobacteria.Deltaproteobacteria.Myxococcales	4.0598046
GM1	l	Bacteria.Acidobacteria.Solibacteres.PAC000121_o.PAC000121_f.PAC000121_g.HM445442_s	4.0543192
GM1	b	Bacteria.AD3.PAC000071_c.PAC000071_o.PAC000071_f.PAC001876_g	4.0500772
GM2	d9	Bacteria.Proteobacteria.Betaproteobacteria.Burkholderiales.Burkholderiaceae.Burkholderia	4.0469921
GM3	z	Bacteria.Actinobacteria.Actinobacteria_c.Pseudonocardiales.Pseudonocardiaceae.JF266448_g.JN802363_s	4.0422871
GM1	a	Bacteria.AD3.PAC000071_c.PAC000071_o.PAC000071_f.PAC001876_g.AY913277_s	4.0304835
GM1	c0	Bacteria.Nitrospirae.Nitrospira_c.PAC001956_o.PAC001956_f	4.0283186
GM2	e0	Bacteria.Proteobacteria.Betaproteobacteria.Burkholderiales.Burkholderiaceae	4.0263344
GM3	c5	Bacteria.Planctomycetes.Planctomycetia	4.0240183
GM2	d8	Bacteria.Proteobacteria.Betaproteobacteria.Burkholderiales.Burkholderiaceae.Burkholderia.Burkholderia thailandensis	4.0239661
GM3	c4	Bacteria.Planctomycetes.Planctomycetia.Planctomycetales	4.0200374
GM1	c1	Bacteria.Nitrospirae.Nitrospira_c.PAC001956_o	4.0178095
GM3	c6	Bacteria.Planctomycetes	4.0050660
